# Mitochondrial misreading in skeletal muscle accelerates metabolic aging and confers lipid accumulation and increased inflammation

**DOI:** 10.1261/rna.077347.120

**Published:** 2021-03

**Authors:** Dimitri Shcherbakov, Stefan Duscha, Reda Juskeviciene, Lisa M. Restelli, Stephan Frank, Endre Laczko, Erik C. Böttger

**Affiliations:** 1 Institut für Medizinische Mikrobiologie, Universität Zürich, 8006 Zürich, Switzerland; 2 Division of Neuropathology, Institute of Medical Genetics and Pathology, Basel University Hospital, 4031 Basel, Switzerland; 3 Functional Genomics Center Zurich, ETH Zürich und Universität Zürich, 8057 Zürich, Switzerland

**Keywords:** mitochondria, misreading, skeletal muscle, aging, metabolome

## Abstract

We have recently reported on an experimental model of mitochondrial mistranslation conferred by amino acid exchange V338Y in mitochondrial ribosomal protein MrpS5. Here we used a combination of RNA-seq and metabolic profiling of homozygous transgenic *Mrps5^V338Y/V338Y^* mice to analyze the changes associated with the V338Y mutation in postmitotic skeletal muscle. Metabolome analysis demonstrated enhanced levels of age-associated metabolites in the mutant V338Y animals accompanied by increased glycolysis, lipid desaturation and eicosanoid biosynthesis, and alterations of the pentose phosphate pathway. In addition, transcriptome signatures of aged V338Y mutant muscle pointed to elevated inflammation, likely reflecting the increased levels of bioactive lipids. Our findings indicate that mistranslation-mediated impairment of mitochondrial function affects specific bioenergetic processes in muscle in an age-dependent manner.

## INTRODUCTION

A decline in mitochondrial function has been associated with aging and complex age-related changes in metabolism (Sun et al. 2016; Kauppila et al. 2017). Alterations in mitochondrial function including impaired oxidative phosphorylation, increased oxidative damage, reduced activity of metabolic enzymes, and changes in mitochondrial dynamics and biogenesis have all been linked to various aspects of aging (Nunnari and Suomalainen 2012). It is now widely accepted that mitochondria are not mere energy factories, but play central roles in metabolism and signaling (Finkel 2015; Spinelli and Haigis 2018).

The accumulation of somatic mutations in mitochondrial DNA (mtDNA) in post-mitotic tissues is thought to be a key factor in the pathogenesis of age-related diseases (Wallace 2005). mtDNA encodes for genes of oxidative phosphorylation (OXPHOS) and corresponding mutations result in a decline of OXPHOS activity, ultimately impairing cellular bioenergetic capacity (Wallace 1999). Mice that express a mtDNA mutator phenotype, with a threefold to fivefold increase in the levels of random point mutations in mtDNA, display respiratory chain dysfunction and features of accelerated aging (Trifunovic et al. 2004; Kujoth et al. 2005).

Increased error rates in mitochondrial protein synthesis may have similar effects to that of heightened levels of stochastic mtDNA mutations as both would increase the random incorporation of missense mutations. Towards this end, we recently demonstrated that the amino acid exchange V338Y in the mitochondrial ribosomal protein MrpS5 confers mitoribosomal mistranslation (Akbergenov et al. 2018). The identification of amino acid replacement V338Y in mouse mitochondrial ribosomal protein MrpS5 as ribosomal ambiguity mutation (*ram*) was based on the experimental demonstration that the human homolog V336Y, while not affecting mitochondrial protein synthesis quantitatively, confers incorporation of near-cognate amino acids and stop-codon read-through in *in-organello* translation assays using mitochondria from human HEK293 cells transfected with mutant V336Y *Mrps5* (Akbergenov et al. 2018). Accommodation of mismatched aa-tRNAs by *ram* mutations increases the natural rate of protein synthesis error frequency, resulting in random missense substitutions in primary amino acid sequences. Due to the physical constraints of the mRNA–tRNA interaction, accommodation of mismatched aa-tRNAs is limited to near-cognate tRNAs, leading to missense amino acid substitutions that are conservative in nature because of the way the genetic code is constructed (Woese 1965; Kurland et al. 1996; Zaher and Green 2009). Homozygous knock-in C57/BL6 mice carrying the *Mrps5* V338Y mutation showed impaired oxidative phosphorylation and increased formation of ROS (Akbergenov et al. 2018).

To further dissect the organismal response to increased mitochondrial misreading, we here combined unbiased RNA-seq and metabolome analysis to characterize the response to mitochondrial mistranslation in the skeletal muscle of *Mrps5^V338Y/V338Y^* mutant mice. Our choice of tissue was guided by muscle consisting of post-mitotic cells with high energy demands and vulnerability to defects in mitochondrial function (Johnson et al. 2013; Boengler et al. 2017). We show that mitoribosomal misreading results in pronounced age-related metabolite changes in muscle, which are associated with alterations of the pentose phosphate pathway (PPP), increased glycolysis and enhanced lipid biosynthesis. Further, increased levels of bioactive lipids suggest a possible link to the transcriptome signature of enhanced inflammation observed in aged *Mrps5^V338Y/V338Y^* mutant mice.

## RESULTS

Transcriptomes of skeletal muscle tissue samples from wild-type and *Mrps5^V338Y/V338Y^* mice were resolved using RNA-seq. To study the impact of the V338Y mutation on age-related changes, mice were sampled at 3 and 19 mo of age. Considerable transcriptomic changes were detected by differential gene expression analysis of young (3 mo) and old (19 mo) muscle samples (Supplemental Fig. S1). With a threshold *P*-value <5 × 10^3^ (FDR < 1 × 10^−2^) there were 3343 and 4232 age-regulated transcripts in the wild-type and V338Y mutant data set, respectively. Further analysis revealed the hallmarks of age-associated transcriptomic alterations for both wild-type and mutant animals—a decrease in mRNA transcripts related to gene annotations associated with mitochondrial function, proteasome degradation, metabolism, and protein translation (Supplemental Fig. S2), for clustering of the experimental groups by BGA see Supplemental Figure S3 (Lee et al. 1999; Su et al. 2015; Shavlakadze et al. 2019).

**FIGURE 1.**
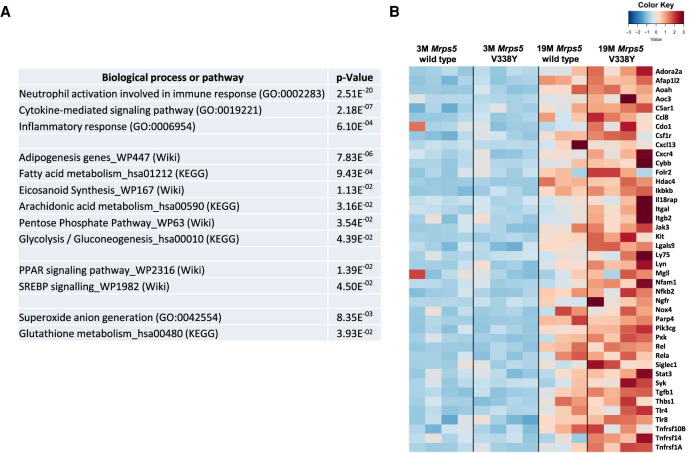
Muscle transcriptome analysis of mice. (*A*) Gene enrichment analysis comparing 19 mo *MrpS5 WT* and *MrpS5* V338Y mutant mice; terms and significance for up-regulated gene transcripts, adjusted *P*-values are shown. (*B*) Expression heatmap of up-regulated genes enriched in GO term “Inflammatory process” across 3 mo and 19 mo muscle samples.

In addition to the characteristic age-associated transcriptome changes, several mutant-specific processes emerged among enriched up-regulated gene transcripts in aged V338Y mice, most prominently multiple terms associated with inflammation and neutrophil activation (Fig. 1A). As evident from the heatmap (Fig. 1B), the expression of inflammation-related transcripts was also elevated in aged wild-type animals, but more prominently in aged V338Y mice. In addition to inflammation, the aged V338Y mutant mice showed enrichment for gene transcripts associated with specific metabolic pathways, that is, adipogenesis, fatty acid (FA) metabolism, eicosanoid synthesis, pentose phosphate shunt, and glycolysis/gluconeogenesis (Fig. 1A).

**FIGURE 2.**
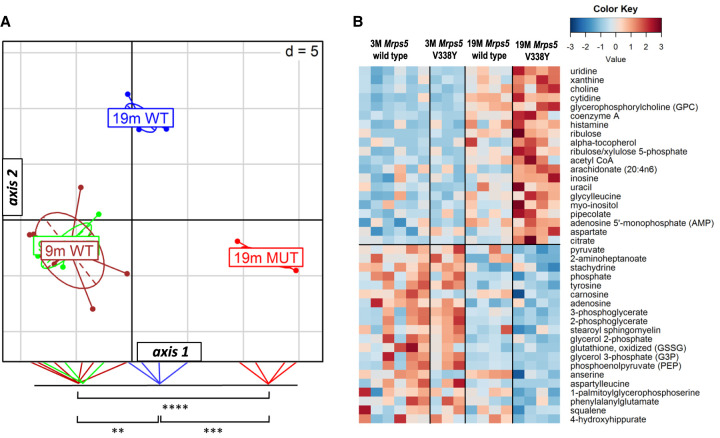
Muscle metabolome analysis. (*A*) Between group analysis (BGA) scatter plot for muscle metabolite profiles (*n* = 6 for 9-mo-old *Mrps5* WT—green; *n* = 3 for 9-mo-old *Mrps5* V338Y—brown; *n* = 4 for 19-mo-old *Mrps5* WT—blue; *n* = 4 for 19-mo-old *Mrps5* V338Y—red). Coordinates of the animals as well as group centers along axis 1 were projected on the *bottom* line of the plot frame and significant group differences are indicated (Welch's *t*-test) (**) *P* = 0.00081, (***) *P* = 0.000078, (****) *P* = 0.000002). (*B*) Heatmap of the top 20 metabolites positively or negatively correlated with horizontal axis (Student's *t*-test for 19 mo *Mrps5* V338Y mice and 19 mo *Mrps5* wild-type animals; *P* < 0.01 for positively correlated metabolites; *P* < 0.05 for negatively correlated metabolites).

To further characterize the mutant MrpS5 V338Y-associated changes in muscle tissue, we performed metabolic profiling for 9 and 19-mo-old mice. For an initial assessment of differences, we performed a between group analysis (BGA). BGA simplifies a data set by reducing its dimensionality and defining axes that best discriminate the groups. We assigned four groups (9 mo wild-type, 9 mo V338Y, 19 mo wild-type, 19 mo V338Y) and plotted them into two-dimensional space using the first two axes. BGA showed a similar metabolic pattern in wild-type and mutant V338Y mice at 9 mo of age (Fig. 2A). At 19 mo, the metabolic profiles of both genotypes were significantly different from the 9 mo animals. In addition, the 19 mo V338Y mice were also distinct from the age matched wild-type controls (*P* < 0.0001). The observed metabolic profile indicates that both age and genotype cause the metabolic differences between these two groups. Extraction of the most differentially up- and down-regulated metabolites associated with the horizontal axis (axis 1) included age-associated metabolites described previously (Supplemental Fig. S4A; Houtkooper et al. 2011), suggesting that axis 1 represents aging, as this axis captures the largest fraction of age-related variance. Calculating the BGA on the basis of the published data set and projecting our animals on this model, corroborated our conclusion that the 19 mo *Mrps5*^V338Y/V338Y^ animals showed enhanced age-associated metabolic changes compared to age-matched wild-type controls (Supplemental Fig. S4B).

The heatmap of the 40 metabolites with the highest correlation to axis 1 (Fig. 2B) indicates reduced levels of late glycolysis intermediates pyruvate, 3-phosphoglycerate and phosphoenolpyruvate in the 19 mo *Mrps5*^*V338Y/V338Y*^ animals, whereas levels of early glycolysis intermediates and lactate remain unchanged (not shown in the heatmap); the same pattern but to a lesser extent was observed in the aged wild-type animals. Transcriptome profiles revealed significant up-regulation (*P* < 0.05) of hexokinases Hk1 and Hk3 (*Hk1/3*), phosphofructokinase (*Pfk*), phosphoglycerate mutase 1 (*Pgam1*), and Enolase 1 (*Eno1*) in 19 mo V338Y animals, indicating increased expression of key enzymes in the glycolysis pathway. Further, the pool sizes of ribose-5-phosphate and ribulose-5-phosphate, key metabolites of the pentose phosphate pathway (PPP) were increased in 19 mo V338Y mutant mice and the key enzymes glucose-6-phosphate dehydrogenase X-linked (*G6pdx*) and transketolase (*Tkt*) were up-regulated at the transcriptome level, as were 6-phosphogluconolactonase (*Pgls*) and phosphogluconate dehydrogenase (*Pgd*) (Supplemental Tables S1, S2), indicative of increased shunting from glycolysis to PPP.

**FIGURE 3.**
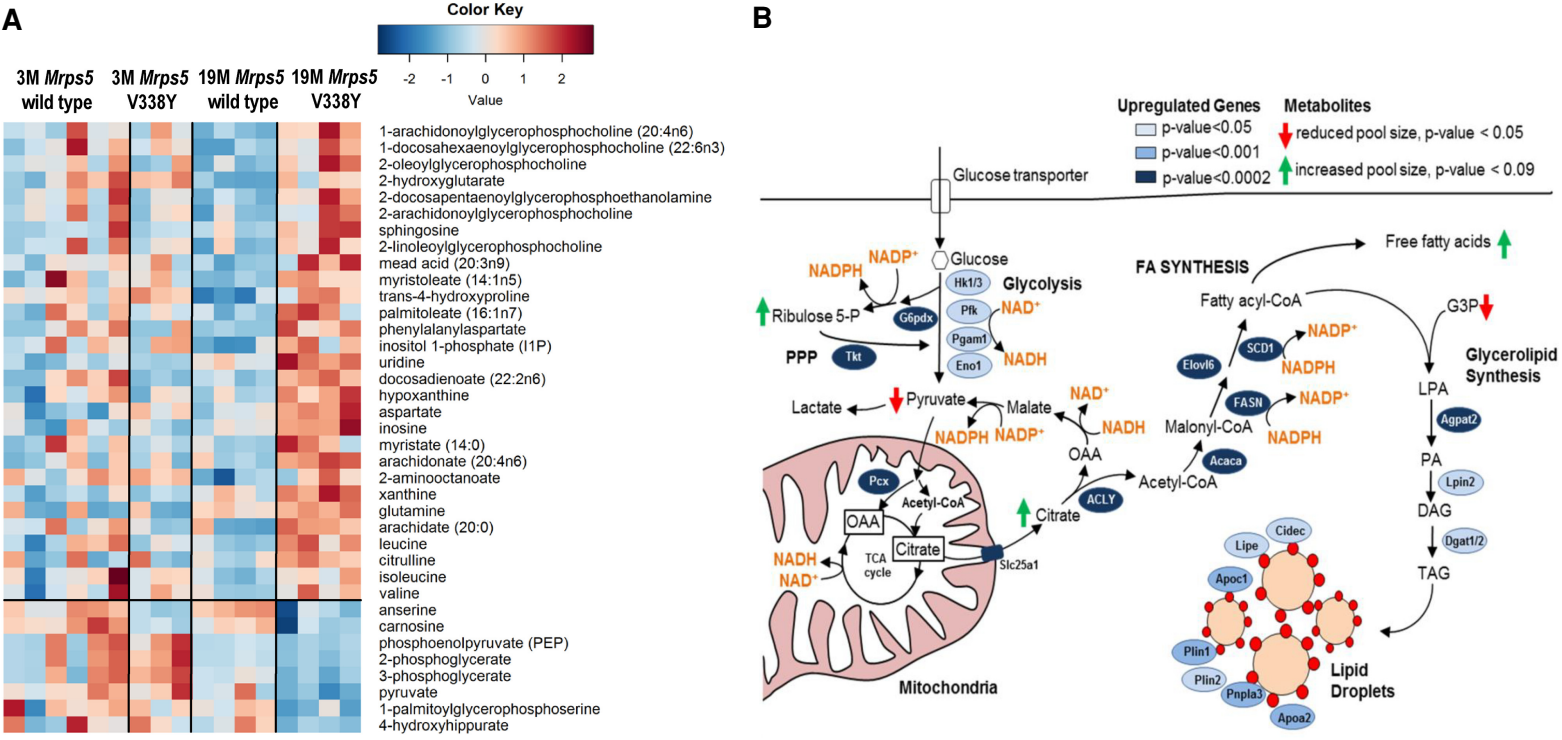
*Mrps5* V338Y mutation-specific changes in 19-mo-old animals. (*A*) Heatmap of metabolites associated with the group of 19 mo *Mrps5* V338Y animals (Students's *t*-test for 19 mo *Mrps5* V338Y mice and 19 mo *Mrps5* wild-type animals; *P* < 0.05). (*B*) Model of metabolite pathways, including enzymes and the electron carrying coenzymes, altered in the *Mrps5* V338Y animals in contrast to wild-type at 19 mo age (PPP) pentose phosphate pathway, (OAA) oxaloacetate, (G3P) glycerol 3-phosphate, (LPA) lysophosphatidic acid, (PA) phosphatidic acid, (DAG) diacylglycerol, (TAG) triacylglycerol.

Based on the BGA, we identified the metabolites which were most increased or decreased in 19 mo *Mrps5*^V338Y/V338Y^ mutant animals and used those to plot a second heatmap (Fig. 3A). The free long chain fatty acids arachidonate, docosadienoate, and arachidate were the most significantly increased metabolites in the mutant animals discriminating between *Mrps5* wild-type and V338Y mutant mice at 19 mo. At the transcript level we observed increased expression of enzymes involved in de novo synthesis of lipids and anaplerotic replenishment of the TCA cycle (Fig. 3B). Overall, saturated, monounsaturated, and polyunsaturated free fatty acids (FAs) accumulated in the aged *Mrps5*^V338Y/V338Y^ mutant animals compared to the 19 mo *Mrps5* wild-type mice (Fig. 3A; Supplemental Table S2). Transcriptome data further suggest increased synthesis of triacylglycerides, lipid droplet formation and lipid droplet coating proteins in V338Y mutant mice as compared to 19 mo wild-type animals (Fig. 3B; Supplemental Table S1). Together with elevated levels of arachidonate and dihomo-linoleate, the two key intermediates of eicosanoid metabolism, we found significant enrichment for gene transcripts involved in eicosanoid biosynthesis (e.g., desaturases, arachidonate-lipoxygenases, glutathione peroxidases, prostaglandin E, and thromboxane A synthases; see Supplemental Table S1). In addition, we observed increased levels of lysophospholipids, a side product of arachidonate release from membrane phospholipids (see Supplemental Table S2), together with increased expression of PLA2ε, the main phospholipase in muscle hydrolyzing membrane phospholipids to produce FAs and lysophospholipids (Ohto et al. 2005). The RNA-seq data were corroborated by quantitative RT-PCR for selected key genes involved in glycolysis, PPP, FA synthesis, glycerolipid synthesis, and lipid droplet formation (Supplemental Fig. S5).

## DISCUSSION

Translational accuracy of cytoribosomal protein synthesis has been linked to organismal longevity, stress response, and metabolic alterations (Azpurua et al. 2013; Kurylo et al. 2018; McMahon et al. 2019), while little is known about mitochondrial protein synthesis in this respect. We here studied the response to mitochondrial mistranslation in *Mrps5^V338Y/V338Y^* mice. Mutation *Mrps5* V338Y is a *ram* mutation, which increases the natural rate of mitochondrial protein synthesis error frequency. We used unbiased RNA-seq to characterize the aging transcriptome in *Mrps5* V338Y mutant and *Mrps5* wild-type skeletal muscle, complemented by metabolome analysis of muscle from young and old animals. Reflecting the previously reported mutation-mediated increase in reactive oxygen species formation (Akbergenov et al. 2018) we find decreased levels of ROS scavengers anserine and carnosine (Fig. 2B), combined with the transcriptomic signatures of increased superoxide anion generation and glutathione metabolism (Fig. 1A). Presumably, the altered PPP activity observed in the V338Y mutants (Supplemental Tables S1, S2) provides the reducing power to maintain the cellular redox potential and antioxidant activity in response to the mitochondrial production of reactive oxygen species (Zhang et al. 2018).

A common feature of aging is the decline of metabolic systems in general and in particular the link to impaired mitochondrial function (Finkel 2015; Sun et al. 2016). RNA sequencing of muscle confirmed the view of metabolic decline that is thought to underlie part of the aging process. Further metabolomic analyses suggested an enhanced age-associated metabolome profile in the V338Y mutants as compared to the wild-type control mice. This conclusion was supported by analysis of an independent set of metabolites characterized in a previous aging study (see Supplemental Fig. S4B; Shavlakadze et al. 2019). While the mutants essentially showed the same age-associated metabolic changes as the wild-type animals, the effect was significantly more pronounced. In normal aging, these changes are linked to a shortage of NAD+, that is, the main shuttle of electrons to the electron transport chain (Titov et al. 2016; Zhang et al. 2016). Our data indicate that mitochondrial mistranslation aggravates age-related bioenergetic processes in an age-dependent manner. We attribute the observed metabolic shift to a combination of mutation- and age-mediated impairment in mitochondrial function. Reflecting this age-dependency we find no histopathological abnormalities in muscle tissue of 9-mo-old *Mrps5* V338Y mice.

Mitochondrial misreading in aged muscle comes along with increased expression of FA synthase, a multifunctional enzyme that catalyzes all seven reactions required for de novo lipid biosynthesis. De novo lipid biosynthesis in MrpS5 mutant cells is initiated by the ATP citrate lyase (Acly) with increased glycolysis and alterations of the pentose phosphate pathway providing the NADPH required for lipogenesis. Acly is a cytosolic enzyme that catalyzes the formation of mitochondria-derived citrate into oxaloacetate and acetyl CoA, which are used as building blocks for lipid synthesis reportedly improving mitochondrial function (Das et al. 2015). Increased expression of genes encoding FA activating enzymes (Acsl5) together with lipid esterification proteins (acylglycerol-3-phosphate acetyltransferase, diacylglycerol acyltransferase) and lipogenesis genes (perilipin 1/2, apolipoprotein CI/AII, lipase) further allows for the coordinate synthesis of lipid droplets in the *Mrps5* mutants. Lipid droplet formation, a hallmark of cellular stress, involves a switch in metabolism to glycolysis-dependent ATP generation and lipid biogenesis (Olzmann and Carvalho 2019). In addition to providing an important source of energy, lipid droplets are hypothesized to serve a protective role under conditions of hypoxia and cellular stress by gathering free fatty acids to protect cells against lipotoxicity (Bozza and Viola 2010; Olzmann and Carvalho 2019). Disruption of mitochondrial function by depleting mitochondrial DNA augments the formation of lipid droplets, causing transcriptional activation of FA biosynthesis and metabolic reprogramming to glycolysis (Lee et al. 2013). In addition to increased FA synthesis, impaired mitochondrial respiration has been shown recently to trigger fatty acid desaturation for glycolytic NAD+ recycling (Kim et al. 2019). Our findings of increased glycolysis together with increased expression of fatty acid desaturases (FADS1/2) and heightened levels of unsaturated and polyunsaturated fatty acids would fit well into a scenario of glycolytic NAD+ regeneration by fatty acid desaturation.

While the complex signaling pathways which mediate this metabolic shift in response to mitochondrial misreading are outside the scope of this manuscript, we note that similar metabolic profiles, characterized by increased FA synthesis and expression of genes implicated in lipogenesis together with an enhanced glucose flux through the PPP, have been associated with peroxisome proliferator-activated receptor coactivator 1α (PPARγC-1α) and sterol regulatory element binding protein (SREBP) (Düvel et al. 2010; Summermatter et al. 2010). Interestingly, we find in our mutants a significant enrichment for gene transcripts annotated as PPAR and SREBP signaling pathways, respectively (Fig. 1A).

In addition to the metabolic alterations, V338Y mutant muscle from 19-mo-old mice shows the transcriptomic signature of inflammation. Chronic inflammation is one of the hallmarks of aging and considered to be of multifactorial origin (Ferrucci and Fabbri 2018). In our *Mrps5* mutant mice, increased inflammation was accompanied by elevated levels of arachidonic acid, dihomo-linoleate and other ω-6-derived polyunsaturated FAs. The polyunsaturated FAs arachidonate and dihomo-linoleate serve as main substrates for the synthesis of eicosanoids (Buczynski et al. 2009), locally acting bioactive signaling lipids that regulate a diverse set of biological processes and that act as highly potent inflammatory mediators (von Moltke et al. 2012; Lämmermann et al. 2013; Dennis and Norris 2015). The increased levels of the substrates arachidonic and dihomo-linoleic acid together with the enhanced expression of key metabolic enzymes of eicosanoid biosynthesis suggest a possible link of metabolic alterations with the transcriptomic signature of enhanced inflammation observed in the aged *Mrps5*^*V338Y/V338Y*^ mutant misreading animals.

## MATERIALS AND METHODS

### Animals

The transgenic *Mrps5^V338Y/V338Y^* mouse strain has been previously described (Akbergenov et al. 2018). Expression levels of *Mrps5* mRNA were comparable in mutant and wild-type mice, indicating that the mutation did not lead to deregulation of *Mrps5* gene expression. Homozygous mutant mice were able to breed and did not show any gross physiological or morphological phenotype. At 3, 9, and 19 mo of age animals were sacrificed, and muscle tissue was carefully dissected, snap frozen and stored at −80°C.

### RNA extraction

For the analysis of quadriceps muscle the following mice were used: four 3-mo-old female animals of *Mrps5^WT/WT^*, four 3-mo-old female animals of *Mrps5^V338Y/V338Y^* three 19-mo-old female animals of *Mrps5^WT/WT^*, and four 19-mo-old animals of *Mrps5^V338Y/V338Y^*. RNA was extracted using TRIzol reagent (Invitrogen) according to the manufacturer's instructions. The quality of the isolated RNA was assessed using a Qubit (1.0) Fluorometer (Life Technologies) and a Bioanalyzer 2100 (Agilent). Only those samples with a 260 nm/280 nm ratio between 1.8–2.1 and a 28S/18S ratio within 1.5–2 were further processed, all samples used for RNA sequencing had RIN (RNA Integrity Number) ≥ 7.5.

### cDNA library preparation and sequencing

RNA sequencing (RNA-seq) was performed at the UZH/ETH Functional Genomics Center Zurich (FGCZ) according to the Illumina RNA sequencing protocol. The TruSeq Stranded mRNA Sample Prep Kit (Illumina) was used in the succeeding steps. Briefly, total RNA samples (100–1000 ng) were ribosome depleted and then reverse-transcribed into double-stranded cDNA with actinomycin added during first-strand synthesis. The cDNA samples were fragmented, end-repaired and polyadenylated. TruSeq adapters containing the index for multiplexing were ligated to the fragmented DNA samples. Fragments containing TruSeq adapters on both ends were selectively enriched with PCR. The quality and quantity of the enriched libraries were validated using Qubit (1.0) Fluorometer and the Caliper GX LabChip GX (Caliper Life Sciences). The product was a smear with an average fragment size of approximately 360 bp. The libraries were normalized to 10 nM in Tris-Cl 10 mM, pH 8.5 with 0.1% Tween 20. The TruSeq SR Cluster Kit v4-cBot-HS (Illumina) was used for cluster generation using 8 pM of pooled normalized libraries on the cBOT. Sequencing was performed on the Illumina HiSeq 2500 single end 126 bp using the TruSeq SBS Kit v4-HS (Illumina).

### Transcriptome data analysis

The quality of the reads was assessed using FastQC (Babraham Bioinformatics [http://www.bioinformatics.babraham.ac.uk/projects/fastqc/]) and potential contaminations were evaluated with FastQ Screen (Babraham Bioinformatics [http://www.bioinformatics.babraham.ac.uk/projects/fastq_screen/]) using bowtie2 v. 2.1.0 (Langmead and Salzberg 2012) default parameters. Quantification of gene expression was performed using the RSEM package (version 1.2.18) (Li and Dewey 2011) mapping against the Ensembl 75 annotations derived from the mouse genome assembly GRCm37. Genes not present (<10 counts per gene) in at least 50% of samples from one condition were discarded from further analyses. Differential gene expression analysis between sample groups of interest was performed using the R/bioconductor package edgeR (Robinson et al. 2010). Differences in gene expression levels were calculated as log_2_ fold changes, resulting *P*-values were adjusted using Benjamini–Hochberg multiple test correction (Benjamini and Hochberg 1995). An adjusted *P*-value <0.05 was considered statistically significant; no fold change threshold was applied.

Functional annotation of differentially expressed genes and pathway enrichment analysis were performed using online biological information tool EnrichR (http://amp.pharm.mssm.edu/Enrichr/) (Kuleshov et al. 2016) that integrates several biological databases and provides a comprehensive set of functional annotation information on genes and proteins. The databases KEGG (Kyoto Encyclopedia of Genes and Genomes, http://www.genome.jp/kegg/pathway.html) (Kanehisa et al. 2019), WP (WikiPathways, https://www.wikipathways.org) (Slenter et al. 2018), GO (Gene Ontology enrichment, http://www.geneontology.org) (Harris et al. 2004) from the EnrichR were used for the analysis. The Benjamini–Hochberg false discovery rate (FDR) procedure was applied to pathway analysis as a correction for multiple testing. Pathways or GO terms with FDR corrected *P*-value (adjusted *P*-value) <0.05 were considered statistically significant. GO terms with adjusted *P*-value <0.05 were subjected to the REVIGO web page (Supek et al. 2011) for removal of redundant GO terms from the results.

### qRT-PCR

To confirm the results of transcriptome analysis, selected genes were subjected to qRT-PCR (for list of genes and primers see Supplemental Table S3). RNA samples were reverse transcribed into cDNA using the High Capacity RNA-to-DNA Kit (Applied Biosystems). cDNA was analyzed by real-time quantitative PCR (qPCR) using an ABI 7500 Fast Real Time PCR system (Applied Biosystems) and a pair of gene-specific primers for each selected gene. qPCR was performed in triplicates using EvaGreen Mix (Bio&SELL) and 20 ng of cDNA per reaction. The transcript levels of the examined genes were normalized to the geometric mean of four housekeeping genes (*GAPDH, Dync1h1, Actb, Rpl41*) (Thomas et al. 2014) from the same sample used as an internal reference and the fold change of mutant relative to the WT mice was calculated as 2^−ΔΔCT^ (Pfaffl 2004). Statistical analysis was performed with GraphPad Prism 5.0 software, unpaired Student's *t*-test was used to estimate significance.

### Metabolome analysis

For metabolome analysis of quadriceps muscle, the following mice were compared: six 9-mo-old female animals of *Mrps5^WT/WT^*, three 9-mo-old female animals of *Mrps5^V338Y/V338Y^*, four 19-mo-old female animals of *Mrps5^WT/WT^* animals, and four 19-mo-old female animals of *Mrps5^V338Y/V338Y^* (Supplemental Data S1). Metabolome analysis was performed by Metabolon (USA). In brief, samples were prepared by a proprietary series of organic and aqueous extractions in order to remove proteins and to recover the maximum amount of small molecules. The extracted samples were split in equal parts and analyzed via GC–MS or LC–MS/MS. For the LC–MS/MS two equal parts were analyzed in the positive (acidic solvent) and in the negative (basic solvent) ionization mode. Samples for GCMS were bistrimethyl-silyl-trifluoroacetamide derivatized and were run with a 5% diphenyl/95% dimethyl polysiloxane fused silica column.

Metabolome data sets were analyzed by between group analysis (BGA) and random forest (RF) algorithm using the made4 or the RRF package (bioconductor.org) in R, respectively. Normalized MS intensity data were used for RF and BGA analyses, the latter based on Principal Component Analysis (PCA). BGA was applied to a data set consisting of 230 metabolites. Results of the BGA were visualized by a scatter plot for the first two axes of the BGA. The criteria for metabolites being correlated to a group was a maximal angle of 18° (cos18 = 0.95 correlation coefficient) of the metabolite's vector to the vector of the center of the corresponding group. Coordinates were used to calculate significance levels between groups by applying Welch's *t*-test. A common data set of 88 metabolites derived from the comparison of a published data set (Shavlakadze et al. 2019) and our data set was used to compare age-associated metabolic patterns. The animals of the published data set were used as training data set (3 mo-old animals *n* = 8, 23-mo-old animals *n* = 7) and a BGA model was calculated, the animals of the present study were used as test data set and projected on the calculated BGA model. The resulting coordinates of individual mice were plotted. For univariate analyses Welch's *t*-test and two-way ANOVA were used.

### Histopathology

An amount of 4 µm sections of formalin-fixed, paraffin-embedded hindlimb skeletal muscle (*M. soleus*, *M. tibialis* anterior) were subjected to hematoxylin-eosin staining. In addition, frozen sections (4 µm) of hindlimb skeletal muscle were subjected to histochemical stains to screen for signs of mitochondrial damage (COX, SDH, NADH, modified Gomori trichrome), abnormal fibre typing and atrophy (ATPases pH 4.2/pH 4.6/pH 9.4), dysregulated autophagic flux (acid phosphatase), pathological accumulation of glycogen (PAS), and neural lipids (ORO). Comparative histopathological analyses of mutant and age-matched wild-type controls at age 9 mo (*n* = 3 per group) did not reveal any differences. In particular, no histological signs of inflammation (e.g., lymphocytic infiltrates), mitochondrial damage, dysregulated autophagic flux, abnormal accumulation of glycogen, or neural lipids were evident.

## DATA DEPOSITION

Transcriptome data are available in the Gene Expression Omnibus (GEO), accession number GSE107520, token utwbackwrzmdxip. Metabolome data have been deposited in EMBL-EBI MetaboLights database and are accessible through accession number MTBLS1594.

## SUPPLEMENTAL MATERIAL

Supplemental material is available for this article.

## Supplementary Material

Supplemental Material
