# Supplementary material for: Mitochondrial misreading in skeletal muscle accelerates metabolic aging and confers lipid accumulation and increased inflammation
Source: RNA. 2021 Mar;27(3):265–72. doi: 10.1261/rna.077347.120 (PMC7901843; doi:10.1261/rna.077347.120)
Supplement: Supplemental Material [file supp_27_3_265__index.html]

Mitochondrial misreading in skeletal muscle accelerates metabolic aging and confers lipid accumulation and increased inflammation — Mitochondrial misreading in skeletal muscle accelerates metabolic aging and confers lipid accumulation and increased inflammation — Supplemental Material 

# Mitochondrial misreading in skeletal muscle accelerates metabolic aging and confers lipid accumulation and increased inflammation

## Supplemental Material

- Supplemental\_Figures.pdf
- Supplemental\_Tables.docx
