# Supplementary material for: Mitochondrial misreading in skeletal muscle accelerates metabolic aging and confers lipid accumulation and increased inflammation
Source: RNA. 2021 Mar;27(3):265–72. doi: 10.1261/rna.077347.120 (PMC7901843; doi:10.1261/rna.077347.120)
Supplement: Supplemental Material [file supp_077347.120_Supplemental_Figures.pdf]

Supplementary Figures

Mrps5$^{WT/WT}$ muscle RNA-seq

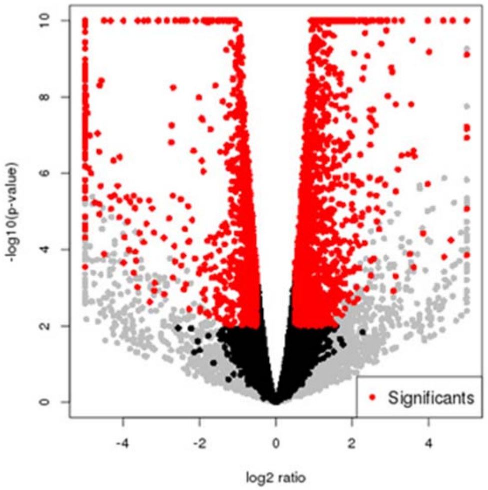

	fc >= 1	fc >= 1.5	fc >= 2
**p < 0.1**	6611	3430	1252
**p < 0.05**	5564	3235	1236
**p < 0.01**	3853	2736	1113
**p < 0.005**	3343	2576	1064
**p < 0.001**	2426	2154	927
**p < 1e-05**	1155	1155	655
**p < 1e-07**	590	590	456
**p < 1e-10**	268	268	264

Mrps5$^{V338Y/V338Y}$ muscle RNA-seq

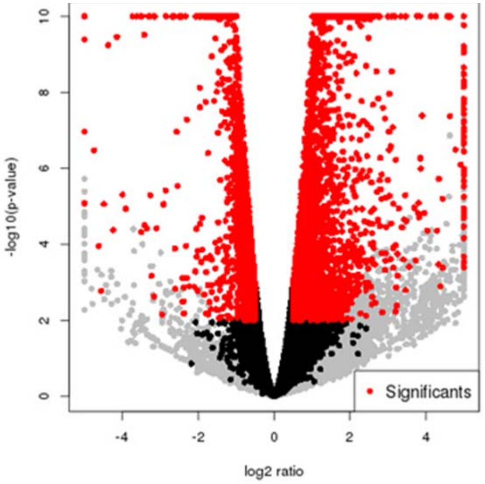

	fc >= 1	fc >= 1.5	fc >= 2
**p < 0.1**	7645	4722	1955
**p < 0.05**	6668	4453	1910
**p < 0.01**	4852	3797	1665
**p < 0.005**	4232	3538	1554
**p < 0.001**	3136	2949	1324
**p < 1e-05**	1412	1412	874
**p < 1e-07**	716	716	605
**p < 1e-10**	299	299	298

Figure S1. RNAseq analysis of Mrps5$^{WT/WT}$ and Mrps5$^{V338Y/V338Y}$ skeletal muscle from mice 3 months and 19 months age. Volcano plots showing fold change ($\log_2$ ratio) and significance of differential expression ($-\log_{10}$(p-Value)) for each gene in aging wild-type (top) and aging mutant (bottom) mice. Each dot represents a single gene. Red dots are significantly regulated genes ($p<0.01$, $\log_2$ ratio>0.5). Tables showing number of regulated genes at different levels of stringency, red frame marks a set of genes that have been chosen for gene enrichment analysis.

A

Biological process	p-Value
Electron Transport Chain WP295	$7.97E^{-46}$
Oxidative phosphorylation WP1248	$1.72E^{-24}$
Proteasome Degradation WP519	$5.27E^{-12}$
Amino Acid metabolism WP662	$2.17E^{-09}$
Glycolysis and Gluconeogenesis WP157	$4.42E^{-08}$
TCA Cycle WP434	$5.48E^{-08}$
Mitochondrial LC-Fatty Acid Beta-Oxidation WP401	$5.34E^{-06}$
Translation Factors WP307	$5.82E^{-05}$
Eukaryotic Transcription Initiation WP567	$8.43E^{-05}$

B

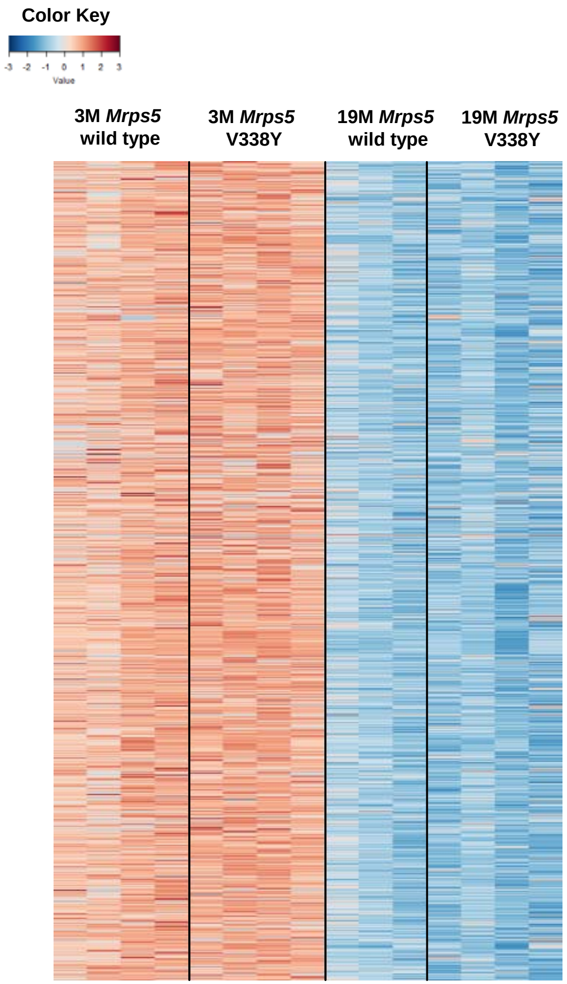

Figure S2. RNAseq analysis of $Mrps5^{WT/WT}$ and $Mrps5^{V338Y/V338Y}$ skeletal muscle from mice 3 months and 19 months age. (**A**) Terms and significances for downregulated gene transcripts (Wiki Pathways; 19m *vs* 3m mice); adjusted p-values are shown. (**B**) Heatmap of genes significantly (p<0.05) down-regulated during aging (19m *vs* 3m mice).

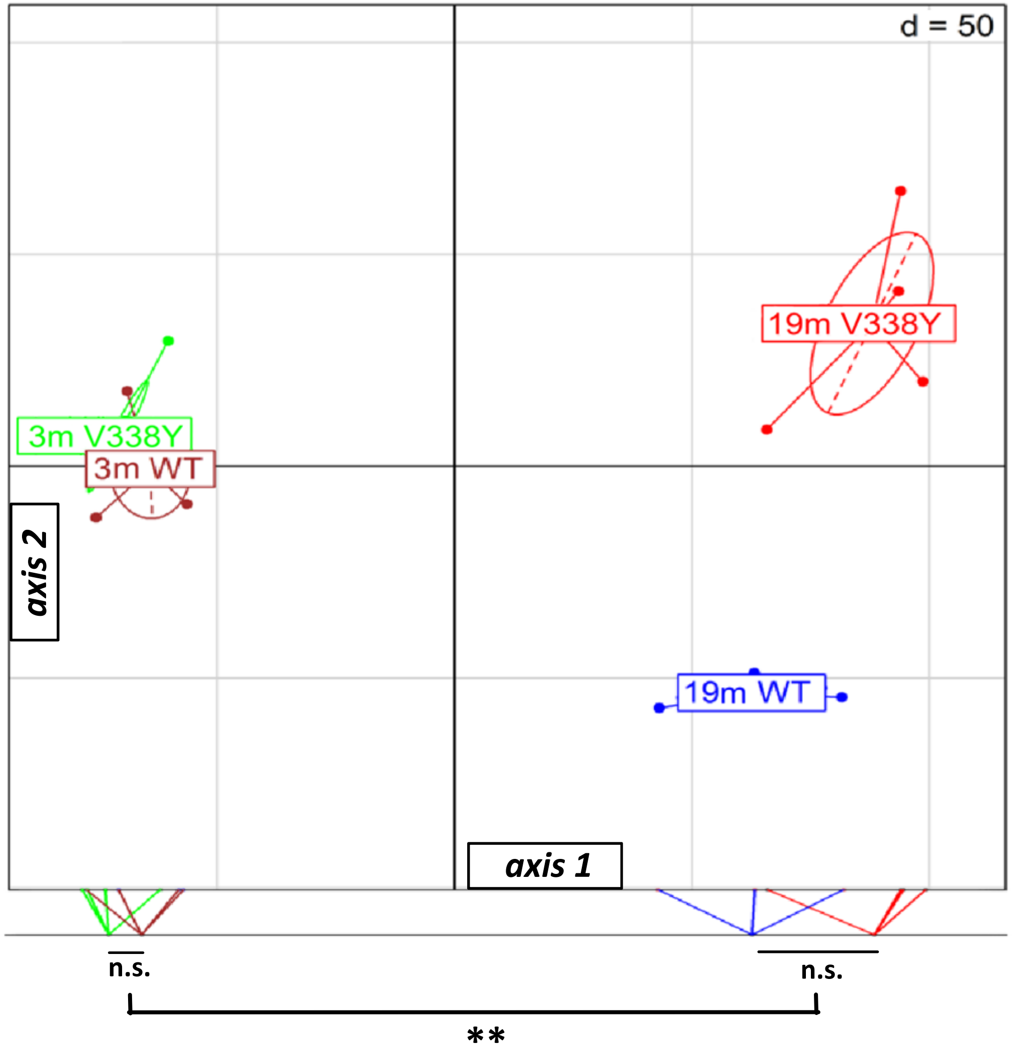

Figure S3. Between group analysis (BGA) plot of muscle samples based on overall gene expression profiles of four groups: 3 months wild-type (brown), 3 months V338Y mutant (green), 19 months wild-type (blue) and 19 months V338Y (red) (Welch's t-test; n.s. - not significant, **P=0.0032).

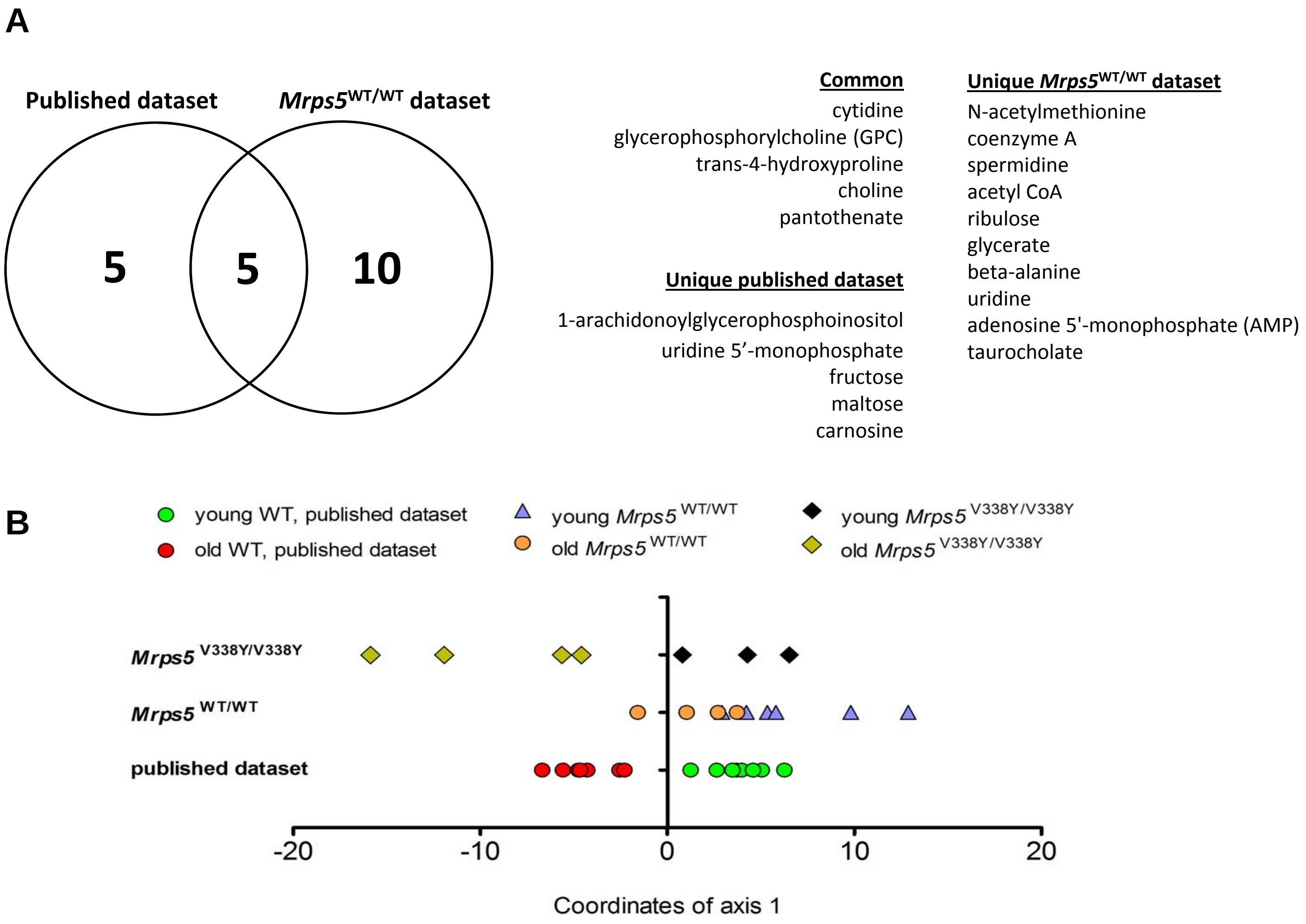

Figure S4. Age-associated metabolites. (**A**) Comparison of 3 months (n=8) and 23 months (n=7) C57/Bl6 mice of published dataset (Houtkooper et al., 2011) with 9 (n=6) and 19 (n=4) months old Mrps5$^{WT/WT}$ mice of the present study. The top 10 age-associated Random Forest (RF) selected metabolites of the published dataset were compared with the top 15 age-associated RF selected metabolites of Mrps5$^{WT/WT}$. Free fatty acids were removed from the comparison, as in the published dataset mice were starved overnight, while in the present study mice were fed *ad libitum* before euthanasia. (**B**) Analysis of common aging pattern between published study (Δold-young = 20 months) and the present study (Δold-young = 10 months). The dataset consisted of 88 common metabolites between the published and the present dataset. BGA of the published mouse dataset was calculated (training dataset). Mice of the present study were used as a test dataset, superimposed in the BGA and coordinates of axis 1 were plotted.

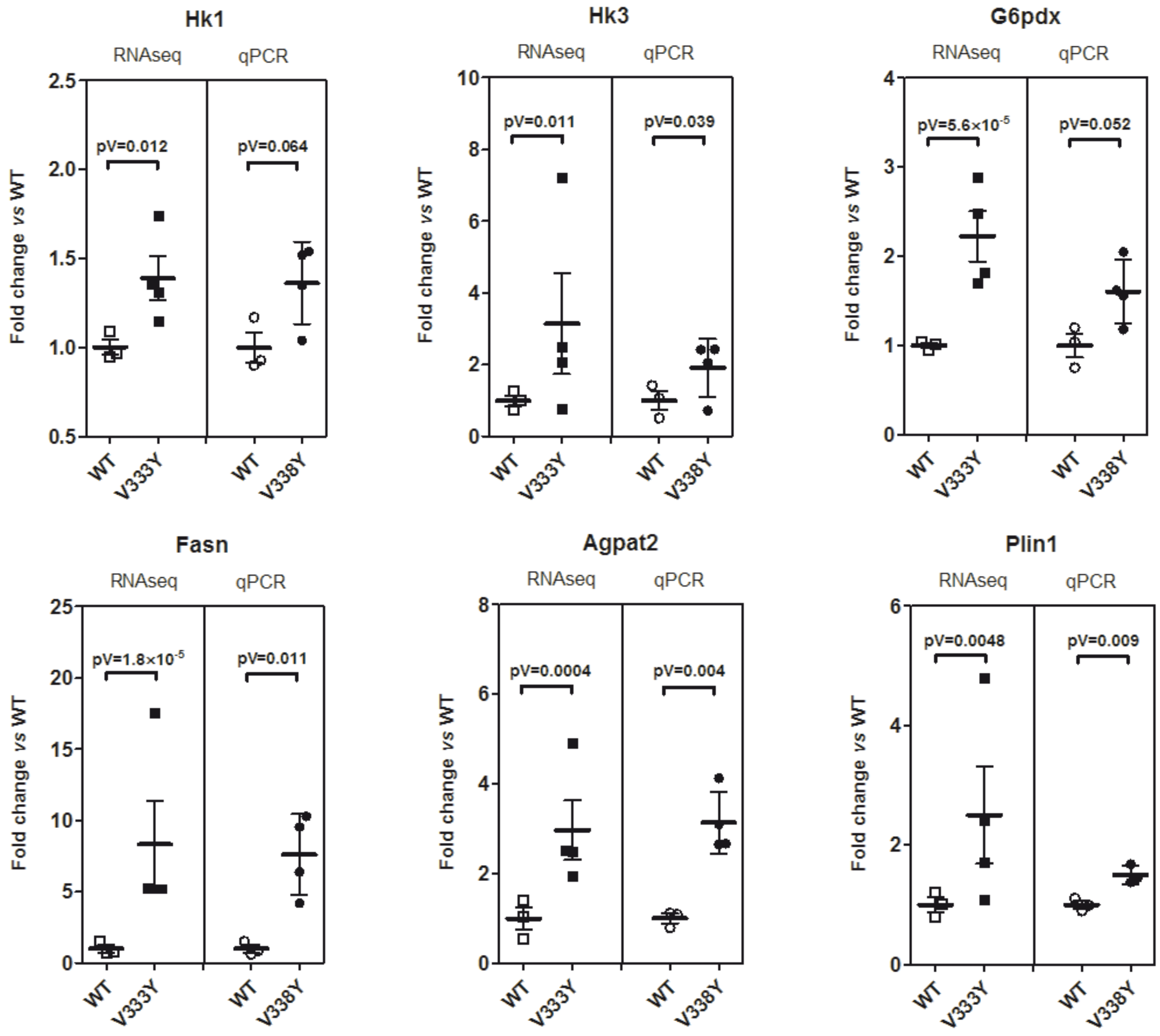

Figure S5. qPCR verification of the RNAseq expression profile of selected genes in the Mrps5$^{WT/WT}$ and Mrps5$^{V338Y/V338Y}$ skeletal muscle from mice of 19 months age. RNAseq data are shown for comparison, corresponding p-values are indicated. Error bars represent SEM.
