# Supplementary material for: Mitochondrial misreading in skeletal muscle accelerates metabolic aging and confers lipid accumulation and increased inflammation
Source: RNA. 2021 Mar;27(3):265–72. doi: 10.1261/rna.077347.120 (PMC7901843; doi:10.1261/rna.077347.120)
Supplement: Supplemental Material [file supp_077347.120_Supplemental_Tables.docx]

**Table S1. Regulated genes in 19 month *Mrps5*^V338Y/V338Y^ compared to wild-type mice**

| **Gene name** | | **Fold change**  (19 M *Mrps5*^V338Y/V338Y^ *vs* *Mrps5*^WT/WT^) | **p-Value** |
| --- | --- | --- | --- |
| **Glycolysis** | |  |  |
| hexokinase 1 | Hk1 | 1.38 | 2.96E-02 |
| hexokinase 3 | Hk3 | 3.10 | 5.12E-03 |
| phosphofructokinase | Pfkl | 1.41 | 4.76E-02 |
| phosphoglycerate mutase 1 | Pgam1 | 1.96 | 1.41E-02 |
| enolase 1 | Eno1 | 1.41 | 1.83E-02 |
|  | | | |
| **Pentose Phosphate Pathway (PPP)** | |  |  |
| glucose-6-phosphate dehydrogenase | G6pdx | 2.20 | 2.69E-06 |
| phosphogluconate dehydrogenase | Pgd | 1.77 | 7.39E-04 |
| 6-phosphogluconolactonase | Pgls | 1.40 | 4.54E-02 |
| transketolase | Tkt | 3.57 | 5.58E-08 |
|  | |  |  |
| **Fatty Acid Synthesis** | |  |  |
| pyruvate carboxylase | Pcx | 2.00 | 9.59E-05 |
| ATP citrate lyase | Acly | 4.02 | 2.08E-04 |
| acetyl-Coenzyme A carboxylase alpha | Acaca | 3.22 | 3.61E-06 |
| fatty acid synthase | Fasn | 8.24 | 2.72E-09 |
| solute carrier family 25 (mitochondrial carrier, citrate transporter), member 1 | Slc25a1 | 3.84 | 4.29E-07 |
| ELOVL family member 6, elongation of long chain fatty acids | Elovl6 | 9.04 | 4.77E-06 |
| fatty acid desaturase 1/2 | Fads 1/2 | 1.49 | 4.00E-02 |
| stearoyl-Coenzyme A desaturase 1 | Scd1 | 5.81 | 1.29E-05 |
| stearoyl-Coenzyme A desaturase 2 | Scd2 | 1.94 | 4.56E-02 |
|  | |  |  |
| **Glycerolipid synthesis** | |  |  |
| 1-acylglycerol-3-phosphate O-acyltransferase 2 | Agpat2 | 2.94 | 9.77E-06 |
| acyl-CoA synthetase long-chain family member 5 | Acsl5 | 1.50 | 2.90E-03 |
| lipin 2 | Lpin2 | 1.88 | 1.77E-02 |
| diacylglycerol O-acyltransferase 1 | Dgat1 | 1.61 | 8.64E-03 |
| diacylglycerol O-acyltransferase 2 | Dgat2 | 2.29 | 3.17E-03 |
|  | |  |  |
| **Lipid droplets** | |  |  |
| perilipin 1 | Plin1 | 2.49 | 9.55E-04 |
| perilipin 2 | Plin2 | 1.67 | 1.12E-02 |
| cell death-inducing DFFA-like effector c | Cidec | 2.10 | 3.06E-02 |
| apolipoprotein A-II | Apoa2 | 9.10 | 9.23E-04 |
| apolipoprotein C-I | Apoc1 | 3.48 | 1.04E-03 |
| patatin-like phospholipase domain containing 3 | Pnpla3 | 1.94 | 9.49E-04 |
| lipase, hormone sensitive | Lipe | 1.85 | 3.68E-02 |
|  |  |  |  |
| **Eicosanoid biosynthesis** | |  |  |
| arachidonate 5-lipoxygenase | Alox5 | 1.72 | 2.24E-03 |
| arachidonate 15-lipoxygenase | Alox15 | 1.75 | 2.17E-02 |
| arachidonate 12-lipoxygenase | Alox12 | 1.88 | 2.50E-02 |
| prostaglandin E synthase | Ptges | 1.89 | 8.05E-04 |
| prostaglandin E receptor 3 (subtype EP3) | Ptger | 2.12 | 6.99E-03 |
| glutathione peroxidase 1 | Gpx1 | 1.45 | 2.73E-03 |
| glutathione peroxidase 8 | Gpx8 | 1.54 | 1.07E-02 |
| carbonyl reductase 2 | Cbr2 | 1.45 | 4.73E-02 |
| thromboxane A synthase 1 | Tbxas1 | 1.38 | 3.50E-02 |
| glutathione S-transferase | Mgst1/2 | 1.73 | 1.31E-02 |
| phospholipase A2, group IVe | Pla2g4e | 2.05 | 1.97E-04 |

Genes of glycolysis, PPP, FA, TAG, and eicosanoid synthesis with a p-value ≤ 0.05 are shown. P-value was calculated using differential gene expression analysis (see Supporting Information Methods) by comparing 19 month *Mrps5*^V338Y/V338Y^ and the aged matched wild-type.

**Table S2. Metabolites with different pool sizes in 19 month *Mrps5*^V338Y/V338Y^ compared to wild-type mice**

| **Metabolite** | **Fold change**  (19 M *Mrps5*^V338Y/V338Y^ *vs Mrps5*^WT/WT^) | **p-Value**  (Student t-Test) |
| --- | --- | --- |
| **Glycolysis** |  |  |
| 3-phosphoglycerate | 0.62 | 0.007 |
| 2-phosphoglycerate | 0.66 | 0.015 |
| phosphoenolpyruvate (PEP) | 0.67 | 0.016 |
| pyruvate | 0.57 | 0.044 |
|  |  |  |
| **Pentose Phosphate Pathway (PPP)** |  |  |
| ribulose 5-phosphate | 1.61 | 0.062 |
| ribose 5-phosphate | 1.94 | 0.063 |
| ribose | 1.85 | 0.072 |
|  |  |  |
| **FA synthesis** |  |  |
| arachidonate (20:4n6) | 1.49 | 0.003 |
| arachidate (20:0) | 1.32 | 0.019 |
| docosadienoate (22:2n6) | 1.63 | 0.001 |
| docosapentaenoate (n3 DPA; 22:5n3) | 1.25 | 0.141 º |
| docosapentaenoate (n6 DPA; 22:5n6) * | 2.05 | 0.159 º |
| adrenate (22:4n6) | 1.45 | 0.191 º |
| dihomo-linoleate (20:2n6) * | 1.38 | 0.128 º |
| eicosapentaenoate (EPA; 20:5n3) * | 1.68 | 0.077 |
| mead acid (20:3n9) | 2.14 | 0.022 |
| stearidonate (18:4n3) | 1.37 | 0.180 º |
| margarate (17:0) * | 1.20 | 0.097 |
| stearate (18:0) | 1.23 | 0.095 |
| myristoleate (14:1n5) | 2.00 | 0.001 |
| myristate (14:0) * | 1.50 | 0.097 |
| eicosenoate (20:1n9 or 11) * | 1.53 | 0.094 |
| palmitate (16:0) | 1.48 | 0.070 |
| palmitoleate (16:1n7) | 1.90 | 0.019 |
| 15-methylpalmitate * | 1.30 | 0.052 |
|  |  |  |
| **TAG synthesis** |  |  |
| glycerol 3-phosphate | 0.57 | 0.059 |
|  |  |  |
| **Lysophospholipids** |  |  |
| 1-arachidonoylglycerophosphocholine (20:4n6) | 4.51 | 0.01 |
| 1-docosahexaenoylglycerophosphocholine (22:6n3) | 4.38 | 0.01 |
| 2-docosapentaenoylglycerophosphoethanolamine | 3.05 | 0.01 |
| 2-oleoylglycerophosphocholine | 3.64 | 0.02 |
| 2-linoleoylglycerophosphocholine | 2.55 | 0.05 |
| 2-arachidonoylglycerophosphocholine | 3.04 | 0.05 |
| 1-stearoylglycerophosphocholine (18:0) | 3.53 | 0.08 |
| 1-linoleoylglycerophosphocholine (18:2n6) | 2.28 | 0.09 |
| 2-docosapentaenoylglycerophosphocholine (22:5n3) | 2.66 | 0.09 |

Metabolites of glycolysis, PPP, glycerophospholipids, FA and TAG synthesis with p-values ≤ 0.1 (Student’s t-test) are shown.

* described aging markers

º these metabolites with p-values > 0.1 were included for completion of pathway analysis

**Table S3. Primers used for real-time quantitative PCR of target and house-keeping genes**

| **Gene symbol** | **Gene name** | **Primers 5’-3’** |
| --- | --- | --- |
| *Hk1* | Hexokinase 1 | ACC AAC CCA CAA AAC AAC GC |
|  |  | CCA AGG AAA CAC CAC TCC GA |
| *Hk3* | Hexokinase 3 | GGT TAC TGC TGT TGC TTG TCG |
|  |  | TCC AAT GAG ATT GCT TCG TTC C |
| *G6pdx* | Glucose-6-phosphate dehydrogenase | GGC ATC GGG GAA GGC CAA A |
|  |  | TTA ACG CAA GAG GGC GTG TG |
| *Fasn* | Fatty acid synthase | GGC CCC TCT GTT AAT TGG CT- |
|  |  | GGA TCT CAG GGT TGG GGT TG |
| *Agpat2* | 1-acyl--glycerol-3-phosphate acyltransferase-beta | CAA GGT CGG TCT CTA CTG CG |
|  |  | TTG AAG GAC CGA ACG AAC CA |
| *Plin1* | Perilipin 1 (lipid droplet-associated protein) | CTA GCT GCT TTC TCG GTG TT |
|  |  | CTG CAG AAC TCT CTG GAG CAC |
| *Gapdh** | Glyceraldehyde 3-phosphate dehydrogenase | CAT CAC TGC CAC CCA GAA GAC TG |
|  |  | ATG CCA GTG AGC TTC CCG TTC AG |
| *Dync1h1** | Cytoplasmic dynein 1 heavy chain 1 | CCG GAG CAC TGC ACT TCT AA |
|  |  | GCC AGC GTA AGC AAT GAA GG |
| *Actb** | Beta-actin | CCT CCC TGG AGA AGA GCT ATG |
|  |  | TTA CGG ATG TCA ACG TCA CAC |
| *Rpl41** | Ribosomal protein L41 | GCC ATG AGA GCG AAG TGG |
|  |  | CTC CTG CAG GCG TCG TAG |

* housekeeping genes used as an internal reference
